# Remarks on the Use of Chloroform

**Published:** 1848-02

**Authors:** Laurence Turnbull

**Affiliations:** Philadelphia


*Remarks on the Use of Chloroform.* By Laurence Turnbull, M. D., of Philadelphia.

Just as the majority of the profession had begun to place confidence in the use of sulphuric ether as an agent for the amelioration of the pain of surgical operations, we are again startled with the announcement of a new agent, called Chloroform, by Prof. Simpson, although some experiments were made with it upon animals six months previously, by M. Flourens, of Paris. This desire for change on the part of our profession acts unfavourably upon the mind of the public, and is apt to produce in them a want of confidence, both in the physician and the remedy.

Chloroform had been used many years before Prof. Simpson's discovery, in combination with alcohol; but when perfectly pure, it is free from alcohol or water. It was made pure by Mr. Samuel Guthrie, at the suggestion of Prof. Silliman, but never used in that state. This was in 1832; so that he is entitled to the credit of being one of its discoverers, with Soubeiran of France, and Liebig of Germany. Dumas has determined its composition to be three equivalents of chlorine to one of bicarburet of hydrogen, (being two of carbon and one of hydrogen, or formule,) on account of its being susceptible of conversion by the action of potassa into formic acid; from this it has received the name of Chloroform.

Having obtained a portion of this new "anæsthetic agent" from the laboratory of Messrs. Powers and Weightman, chemists of this city, and also from Mr. E. Roussel, the celebrated perfumer, I found both possessed of the following properties:

The liquids were colourless and very heavy. Their odour very penetrating and sweet, possessing a burning saccharine taste, which remained in the mouth for several seconds, irritating the papillæ of the tongue, and when kept applied to the face for some time, producing redness and afterwards the removal of the cuticle. This Soubeiran attributes to the impurity of the preparation. Their vapour was not as volatile as hydrated ether. Pure chloroform falls to the bottom in water, dissolving slightly in that liquid; but if mixed with alcohol, it unites very readily in all proportions with water. Its spec. grav. is 1.45; the specimen prepared by Messrs. Powers & Weightman being concentrated from strong sulphuric acid, and that by Mr. Roussel from sulphuric acid and chloride of calcium. Sulphuric acid is a good test of its purity. If the colour be changed by the acid, it contains alcohol; but if pure, the acid does not affect its transparency.

I have administered the chloroform in ten cases, but not with the success ascribed to it by Prof. Simpson. In four cases it produced nausea and vomiting, with great debility and prostration of the nervous system—the patient feeling its effects for several hours. In two cases it caused coughing; and in all the quantity required to produce insensibility to pain, was from one hundred and fifty to two hundred drops. In four cases it produced simple intoxication, the pulse varying from one hundred to one hundred and fifty, and when completely under its influence, the pulse was again reduced.

In some remarks made by Prof. Mütter in the Surgical Clinic of Jefferson College, he deprecated the spirit of seeking after a new agent before we had time fully to have investigated the properties of sulphuric ether. As for himself, he fully believed in the efficacy of the sulphuric ether, having given us good evidence of the same by his numerous operations before the class this winter. The Professor also cautioned us to be careful, for he had heard of a case of convulsions in a young gentleman from the inhalation of chloroform; he still, however, intends to use it in private before he administers it in public, being willing to give it a fair trial, as the bad effects may have arisen from the use of an impure article. M. Sédillot relates a case in which the patient appeared perfectly insensible from the use of chloroform; but each time the point of the bistoury touched the affected limb, it was quickly withdrawn. Ether was then tried, and all traces of muscular contraction disappeared. According to M. Amussat, its effects are the same as ether upon the arterial blood, causing it to resemble venous blood.

The methods of preparing it are somewhat various. Guthrie obtained it by distilling a gallon from a mixture of three pounds of chloride of lime and two gallons of alcohol, of spec. grav. 0.844, and then rectified by redistillation; first, from a great excess of chloride of lime, and afterwards from sulphuric acid.

Soubeiran obtained it by distilling one part of alcohol at 33° with thirty-two parts of liquid chloride of lime, made of one part of solid chloride to five of water. The receiver being surrounded by cold water, and the retort heated till the liquid began to boil, the fire was then removed, and the distillation continued until no more ether passed over. By repeated washings and distillations (the last one from the chloride of calcium) it was obtained in a highly concentrated state. In a note to the Academie des Sciences of Paris, Nov. 29, 1847, M. Soubeiran remarks, that to get rid of any free chlorine, the chloroform should be washed with a solution of the carbonate of soda," in the same manner that we wash sulphuric ether for inhalation by a solution of carbonate of potassa. The free chlorine, I have no doubt, by combining with the hydrogen of the air, generates hydrochloric acid, which is one of the causes of the reddening and desquamation of the cuticle, or we may even have free sulphuric acid if concentrated from that substance, and not neutralized by an alkali.

Thenard recommends it to be obtained by dissolving one part of chloride of lime in three parts of water, decanting the solution, adding from one-fifth to one-tenth of alcohol, and distilling from a retort sufficiently capacious to guard against the overflowing of the materials when they swell. In the receiver water will condense, swimming over an oleaginous liquid. The water is decanted, and the oily liquid being agitated several times with concentrated sulphuric acid, and rectified from finely powdered baryta. This process will furnish pure chloroform, which boils at 142°, and has a spec. grav. of 1.480.

The following is the formula of Dr. Charles T. Jackson, of Boston, a distinguished Chemist, and the discoverer of Etherization, communicated by a letter to Dr. Lewis Roper, an eminent Dentist of this city, who has placed it at our disposal.

"A recent discovery of substituting per chloride of formyle for sulphuric ether, has been made by Prof. Simpson, and I have repeated the experiments with entire success; I made the first pure chloroform that was applied in Boston, and applied it according to Prof. Simpson's directions. Chloroform was, I believe, first made in this country, by Samuel Guthrie, of Sackett's Harbor, and it has been used, as it comes from the still, as chloric ether. (See American Journal Science, vol. xxii., p. 105.) I tried it as mixed with alcohol, and hence failed, but on learning Prof. Simpson's success, I purified some, and then succeeded perfectly in a number of cases, and furnished samples of it to some of the most eminent physicians and dentists of this place. It is an admirable preparation, and very elegant. Your inhaler, reduced to a much smaller size, would answer admirably for its exhibition, for it would save exterior evaporation, by which much of this very volatile and valuable liquid is lost. I have thus far administered it with a small sponge, or a cambric handkerchief. It is prepared by the distillation of very dilute alcohol and hypochlorite of lime. The distilled liquid is then mixed with a large volume of water, and it becomes milky, and the chloroform subsides to the bottom like an oily mass, but heavier than water. The water is decanted or drawn off with a syphon, and the chloroform is obtained nearly pure. Put some concentrated sulphuric acid in a retort, and fix a tube in the tubulure, and attach the retort to an ice cold condensing apparatus, pour in the wet chloroform, and it will immediately distil over without the application of other heat than that generated by the action of the water on the sulphuric acid. If it does not all come over, apply a water bath heat, and you will have pure chloroform, thirty drops of which is a dose for inhalation."

